# The incidence of childhood-onset type 1 diabetes, time trends and association with the population composition in Sweden: a 40 year follow-up

**DOI:** 10.1007/s00125-022-05816-0

**Published:** 2022-10-20

**Authors:** Ingeborg Waernbaum, Torbjörn Lind, Anna Möllsten, Gisela Dahlquist

**Affiliations:** 1 Department of Statistics, Uppsala University, Uppsala, Sweden; 2 Department of Clinical Science, Paediatrics, Umeå University, Umeå, Sweden

**Keywords:** Children, Immigration, Incidence, Time trend, Type 1 diabetes

## Abstract

**Aims/hypothesis:**

During the 1980s and 1990s, the incidence of childhood-onset type 1 diabetes more than doubled in Sweden, followed by a plateau. In the present 40 year follow-up, we investigated if the incidence remained stable and whether this could be explained by increased migration from countries reporting lower incidences.

**Methods:**

We used 23,143 incident cases of childhood-onset type 1 diabetes reported between 1978 and 2019 to the nationwide, population-based Swedish Childhood Diabetes Registry and population data from Statistics Sweden. Generalised additive models and ANOVA were applied to analyse the effects of onset age, sex, time trends and parental country of birth and interaction effects between these factors.

**Results:**

The flattening of the incidence increase seems to remain over the period 2005–2019. When comparing the incidence of type 1 diabetes for all children in Sweden with that for children with both parents born in Sweden, the trends were parallel but at a higher level for the latter. A comparison of the incidence trends between individuals with Swedish backgrounds (high diabetes trait) and Asian backgrounds (low diabetes trait) showed that the Asian subpopulation had a stable increase in incidence over time.

**Conclusions/interpretation:**

In Sweden, the increase in incidence of childhood-onset type 1 diabetes in the late 20th century has been approaching a more stable albeit high level over the last two decades. Increased immigration from countries with lower incidences of childhood-onset type 1 diabetes does not provide a complete explanation for the observed levelling off.

**Graphical abstract:**

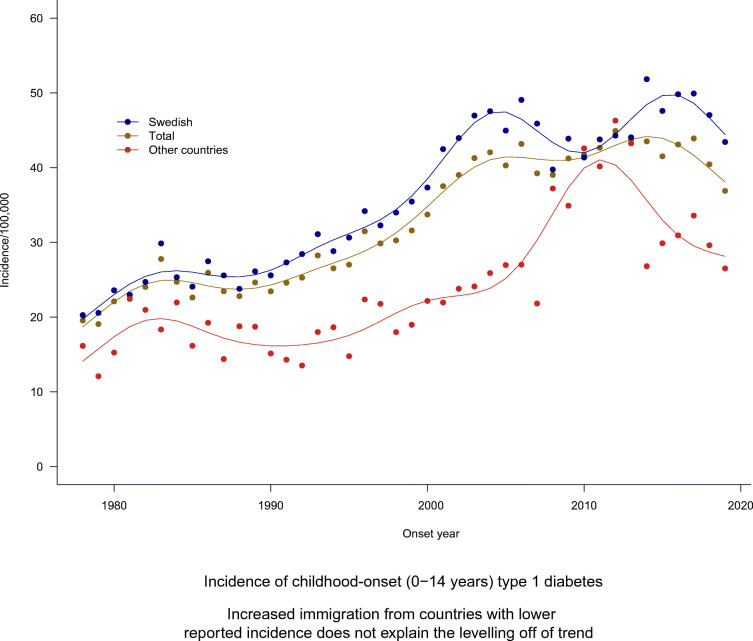

**Supplementary Information:**

The online version of this article (10.1007/s00125-022-05816-0) contains peer-reviewed but unedited supplementary material.

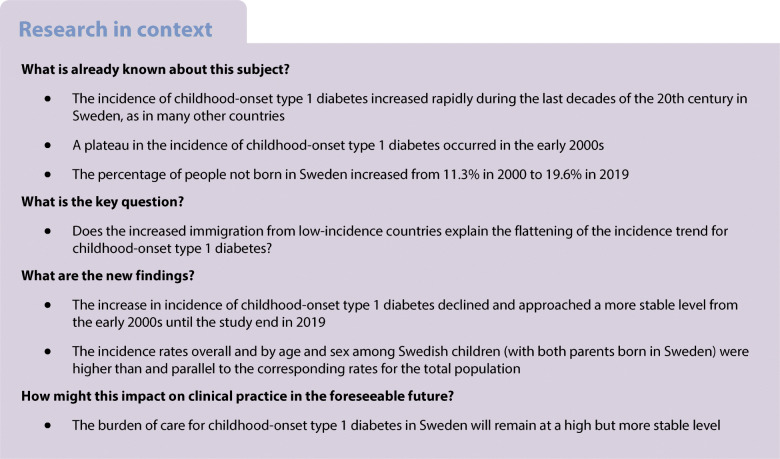



## Introduction

Population-based time trend mapping of disease occurrence is important, including in non-communicable diseases such as type 1 diabetes. Disease occurrence is a basis for generating hypotheses on triggers of clinical onset of the disease and is also an instrument for healthcare planning. Between the early 1980s and the first years of the 21st century, the incidence of childhood-onset type 1 diabetes doubled in Sweden [1, 2]. As many diabetes centres began registering incident cases of this burdensome disease, similar increases in childhood-onset type 1 diabetes were subsequently seen in many countries around the world [3–5]. These rapid changes in incidence could only be explained by changes in environmental risk factors, affecting the autoimmune pathogenesis of the disease [6–10]. The search for such risk factors has been intense and, among them, infectious diseases and lifestyle habits, leading to accelerated child growth and weight gain, have been major foci. The incidence trend is, however, better explained by the latter, as infectious diseases tend to have epidemic patterns of variability.

In the 30 year follow-up of the nationwide Swedish Childhood Diabetes Registry (SCDR), we observed a clear tendency to a plateau in the incidence trend during the years 2002–2007 [2]. Some other population-based registers noticed similar patterns [11, 12] and Finland recently reported a tendency to a decrease in incidence [13], whereas, globally, a majority of study centres still report an increase [5]. Many factors might explain a temporal change in trend and a plateau could be of short duration. It was thus important to follow up, on a nationwide scale, this potentially hopeful change in the diabetes incidence trend in Sweden.

After Finland, Sweden reports the second highest incidence of childhood-onset type 1 diabetes at a national level in international comparisons [3, 4], which suggests a high genetic risk. Studies from the USA have shown that the incidence and prevalence of both type 1 and type 2 diabetes in youth differ among ethnic groups, with the largest prevalence of type 1 diabetes among non-Hispanic white people [14, 15]. One factor that may affect diabetes incidence is migration, which may introduce both changes in the genetic risk in the population and new lifestyle habits. The number of individuals living in Sweden but born, or with parents born, outside Sweden, especially from countries with lower risks of type 1 diabetes, increased rapidly from 11.3% in 2000 to 19.6% in 2019 [16]. This may potentially explain part of the plateau in incidence observed.

We now report the 40 year follow-up of the SCDR of children living in Sweden who developed type 1 diabetes before the age of 15 years.

We analysed the incidence trends by age at onset, sex and parental country of birth to answer the question of whether the plateau in type 1 diabetes incidence, which was observed from 2000 until 2007, was stable during the following 12 years. To remove a possible effect of a changing population composition, we investigated separately children born in Sweden with two parents born in Sweden. Additionally, a subgroup consisting of children born in Asia was also studied.

## Methods

### Study design and participants

The overall study population comprised 23,143 type 1 diabetes cases recorded in the SCDR from January 1978 to December 2019. The childhood-onset incident cases in the SCDR have a high level of ascertainment, the coverage having been estimated to be 96–99% [17]. The SCDR changed the recruitment process in 2010 from a system where paediatric clinics reported their incident cases to the register to a system in which cases are recruited from the Prescribed Drug Register, an official, nationwide register that was started in 2005 and is kept by the Swedish National Board of Health and Welfare [18]. Any child below 15 years of age with at least two filled prescriptions of insulin will be registered in the SCDR. From 1978 to 2009, the SCDR contained only individuals diagnosed with type 1 diabetes in Sweden, whereas since 2010 the register has also contained a small number of children who may have been diagnosed outside Sweden but who receive insulin prescriptions in Sweden. However, 96.1% of cases in the register were born in Sweden (Table 1) and diagnosed in Sweden and thus a maximum of 3.9% may have been diagnosed in their country of birth. The National Board of Health and Welfare provided us with a concordance analysis of the two different sampling methods used in 1978–2009 and 2010–2019. During the 3 year period 2007–2009, 1953 cases were reported from the paediatric clinics and 2011 cases were identified through at least two filled prescriptions of insulin in the Prescribed Drug Register, showing 97.1% agreement between the sampling methods (electronic supplementary material [ESM] Table 1). The very low child mortality rate in Sweden makes under-ascertainment of cases due to deaths in children with un-notified diabetes very unlikely.

**Table 1:** Birth countries of individuals who were diagnosed with type 1 diabetes before 15 years of age in Sweden 1978–2019 and their parents (*n*=23,143)

Birth country	Type 1 diabetes
Country of birth (child)	
Sweden	22,250 (96.1)
Other Nordic countries	107 (0.5)
Other	786 (3.4)
EU25 excluding Nordic countries^a^	40 (0.2)
EU28 excluding Nordic countries^b^	124 (0.5)
Africa	106 (0.5)
Asia	256 (1.1)
North America	26 (0.1)
South America	18 (0.1)
Country of birth (mother)	
Sweden	19,936 (86.1)
Other Nordic countries	903 (3.9)
Other	2304 (10)
EU25 excluding Nordic countries^a^	169 (0.7)
EU28 excluding Nordic countries^b^	214 (0.9)
Africa	426 (1.8)
Asia	687 (3.0)
North America	36 (0.2)
South America	89 (0.4)
Country of birth (father)	
Sweden	19,716 (85.2)
Other Nordic countries	756 (3.3)
Other	2671 (11.5)
EU25 excluding Nordic countries^a^	207 (0.9)
EU28 excluding Nordic countries^b^	205 (0.9)
Africa	493 (2.1)
Asia	684 (3.0)
North America	59 (0.3)
South America	92 (0.4)
Born in Sweden, two parents born in Sweden	18,616 (80.4)

Data are *n* (%), including missing country of birth

^a^EU25: Belgium, Denmark, Germany, Greece, Spain, France, Ireland, Italy, Luxemburg, the Netherlands, Austria, Portugal, Finland, Sweden, UK, Czech Republic, Estonia, Cyprus, Latvia, Lithuania, Hungary, Malta, Poland, Slovenia and Slovakia

^b^EU28: EU25 and Croatia, Romania and Bulgaria

To disentangle the effects of immigration of individuals from low-incidence populations on the incidence trends of type 1 diabetes, we separately investigated a study population of individuals born in Sweden with both parents born in Sweden (referred to as ‘Swedish’, *n*=18,616) compared with the contrasting population, that is, all cases with at least one of the criteria not fulfilled (referred to as ‘Other countries’, *n*=4527). We additionally analysed a subset of the Other countries population, incident cases with at least one parent born in Asia (*n*=586), referred to as ‘Asian’. This was motivated by our aim to follow individuals with a known low genetic risk. The Asian group also represented the most numerous immigrant group (3%) from outside the Nordic countries, although living under the same exposure to societal conditions as the Swedish subgroup [19]. Data for the total population together with the Swedish and Asian subgroups were retrieved from Statistics Sweden.

### Analyses

For the total study population and the Swedish subgroup, we describe the incidence trends for the years 1978–2019 by age at onset and sex to answer the questions stated in the Introduction. Additionally, we contrast the incidence trends in the Swedish and Asian study populations for the years 1980–2019. The incidence rates for the total population and subpopulations defined above were calculated using population data from Statistics Sweden. Incidence rates for the total, Swedish and Asian populations were standardised by age and sex and are provided in an excel file as supplementary material (ESM Table 2).

The statistical analysis used generalised additive models (GAMs) and ANOVA was used to analyse the effects of age at onset, sex, time trends (calendar year) and parental country of birth (Swedish/Other countries) and the interaction effects of calendar year and age at onset. The packages mgcv and modEvA from the statistical software R were used, with the functions gam and D-squared [20].

The incidence of childhood-onset type 1 diabetes was the main outcome variable and we fitted a Poisson model with a non-linear time trend using the package mgcv [20]. Because the outcome was a rate, the log of the population at risk was used as an offset for each time point. The explanatory variables were age at onset, sex, calendar year and the defined country variable (Swedish/Other countries) and the interaction between calendar year and age at onset. Birth countries for all cases and their parents are displayed in Table 1. The graphs of the incidence trends display incidences over the calendar years 1978–2019 and 1980–2019. The solid lines in the figures are predicted cases according to the fitted models. No adjustments were made for multiple testing.

## Results

**Fig. 1:**
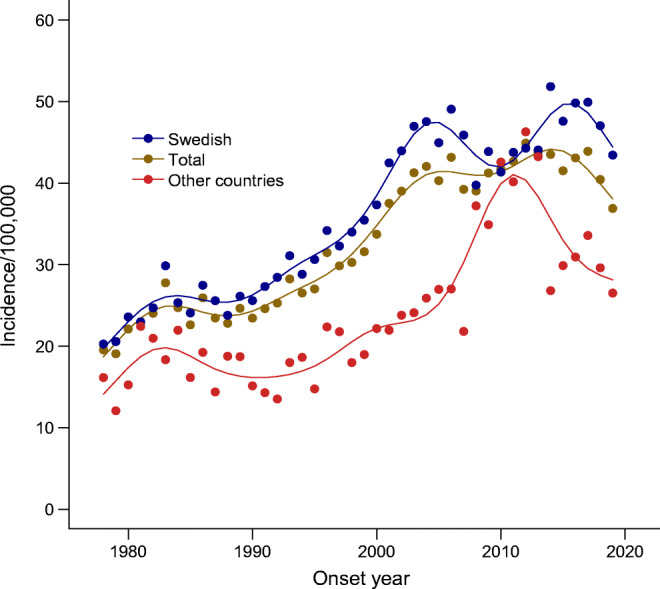
Incidence of type 1 diabetes per 100,000 by calendar years 1978–2019 for the total population of children below 15 years of age in Sweden and the subgroup of the population where the child and both parents were born in Sweden

**Fig. 2:**
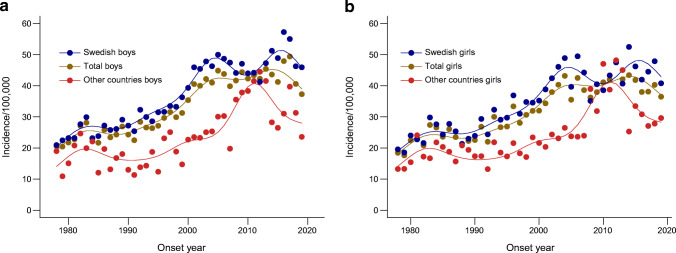
Incidence of type 1 diabetes per 100,000 by calendar years 1978–2019. (**a**) Total and Swedish population of boys. (**b**) Total and Swedish population of girls. Lines represent predicted incidence from GAM model

**Fig. 3:**
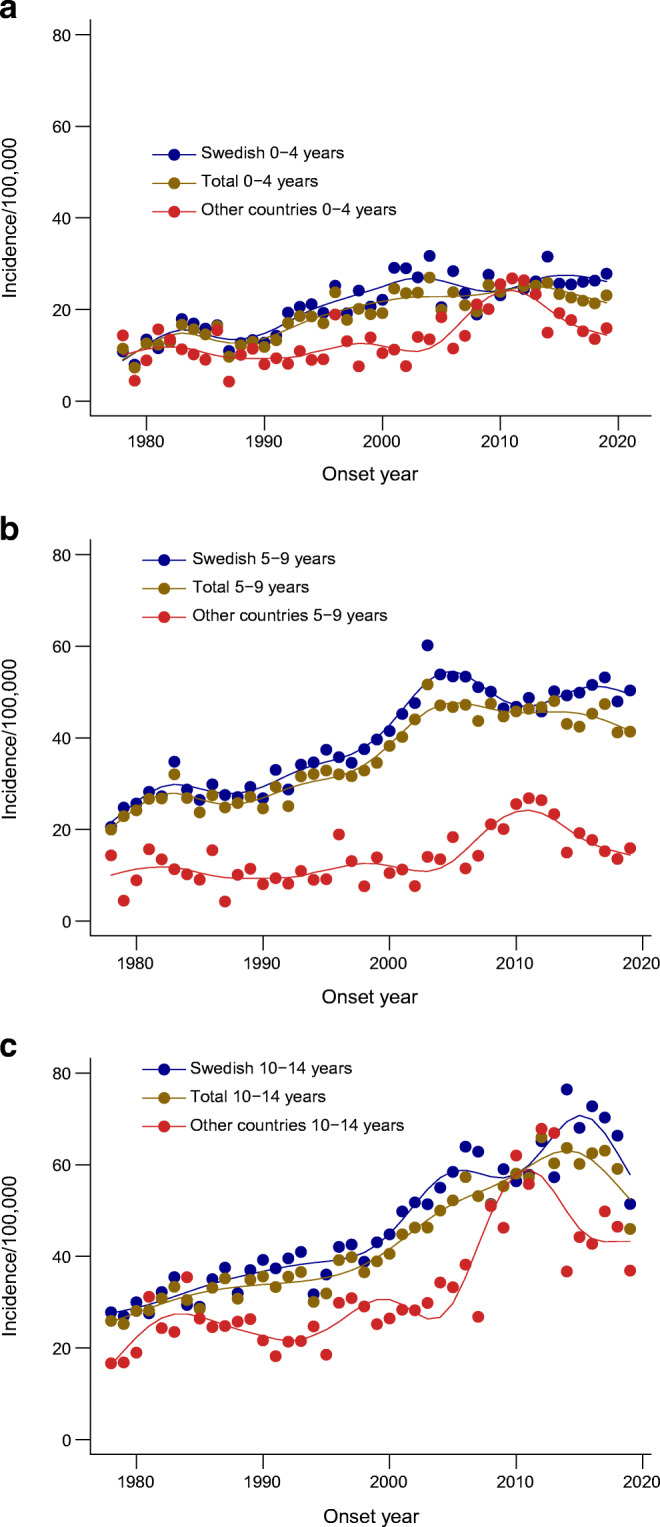
(**a**) Incidence of type 1 diabetes per 100,000 by calendar year for total and Swedish population for age at onset group 0–4 years. (**b**) Incidence of type 1 diabetes per 100,000 by calendar year for total and Swedish population age at onset group 5–9 years. (**c**) Incidence of type 1 diabetes per 100,000 by calendar year for total and Swedish population age at onset group 10–14 years. Lines represent predicted incidence from GAM model

The demographic characteristics of the 23,143 individuals in the study population are described in Table 2 and ESM Table 3, together with the number of person-years at risk. The distributions of individuals according to age at onset for the three age categories 0–4 years, 5–9 years and 10–14 years were 21.3%, 42.3% and 36.4%, respectively. There were slightly more boys (53.1%) than girls (46.9%). Individuals born in Sweden with two parents born in Sweden were in the majority compared with individuals having at least one parent born in another country (80.4% vs 19.6%). The respective age and sex distributions were similar within the subgroups (Table 2), with a slightly higher proportion of boys in the Swedish subgroup. From 1978 to 2000, the incidence trends in the total population and in the Swedish subpopulation showed a steady increase (Fig. 1), but from 2000 the increase in observed cases levelled off until the end of the observation period, when cases again fluctuated. In Figs 1–3, the observed and GAM-predicted incidences for the subgroup Other countries are based on cases and population numbers, subtracting the Swedish subgroup from the population, and amounting to around a quarter of the incident cases in total for the whole time period. For the Swedish boys and girls, the general shapes of the incidence curves were similar to those for the total male and female population, respectively, but at a higher level throughout the observation period and with a larger variation in observed cases after the early 2000s (blue and brown lines in Fig. 2a,b), when two similar cycles appeared, ending with a decrease in both the observed and predicted incidences. Analysis of the incidence trends by age at onset (Fig. 3a–c) shows that the 0–4 years group showed the clearest stabilisation in incidence after the year 2000, both in the total population and among Swedish cases. In the 5–9 years group, there is a similar plateau in the total population after the year 2000, but with a larger variation among Swedish cases. Finally, in the 10–14 years group, there is a continued increase in incidence until around 2015 in both the total population and the Swedish subpopulation, after which the predicted incidences in both groups showed a downward trend, again at a higher level and with a larger variation in the Swedish subpopulation.

**Table 2:** Demographic characteristics of the overall population of children diagnosed with type 1 diabetes below 15 years of age and the subpopulations born in Sweden with both parents born in Sweden (Swedish) and with at least one parent not born in Sweden (Other countries)

Characteristic	Swedish (*n*=18,616)	Other countries (*n*=4527)	Overall (*n*=23,143)
Age categories			
0–4 years	3978 (21.4)	958 (21.2)	4936 (21.3)
5–9 years	7846 (42.1)	1945 (43.0)	9791 (42.3)
10–14 years	6792 (36.5)	1624 (35.9)	8416 (36.4)
Sex			
Girls	8654 (46.5)	2211 (48.8)	10,865 (46.9)
Boys	9962 (53.5)	2316 (51.2)	12,278 (53.1)

Data are *n* (%)

Analysis of the time trend and the associations between incidence and the demographic variables sex, age at onset and parental country of birth in the regression model showed that, besides the known effects of calendar year, age and sex, the defined country indicator had a significant effect and explained 81% of the adjusted deviance, up from 14% when only calendar year, age and sex were included (Table 3).

**Table 3:** ANOVA from nested GAMs with non-linear time trend

Model^a^	*p* value	Adjusted deviance explained (*D*^2^ adj)^b^
1. Year		0.09
2. Year + age at onset	<0.001	0.14
3. Year + age at onset + sex	0.28	0.14
4. Year + age at onset + sex + country	<0.001	0.81
5. Year + age at onset + sex + country + year × age at onset	0.001	0.81

^a^The models are evaluated with the response variable being the incidence of type 1 diabetes and the log of population at risk as the offset. For the time trend, we use a smooth nonparametric term; linear coefficients are assumed for age, sex and interaction terms

^b^*D*^2^ adj is the equivalent of *R*^2^ adjusted for generalised linear models, taking into account the number of observations and the number of predictors, and thus permitting direct comparison among models

**Fig. 4:**
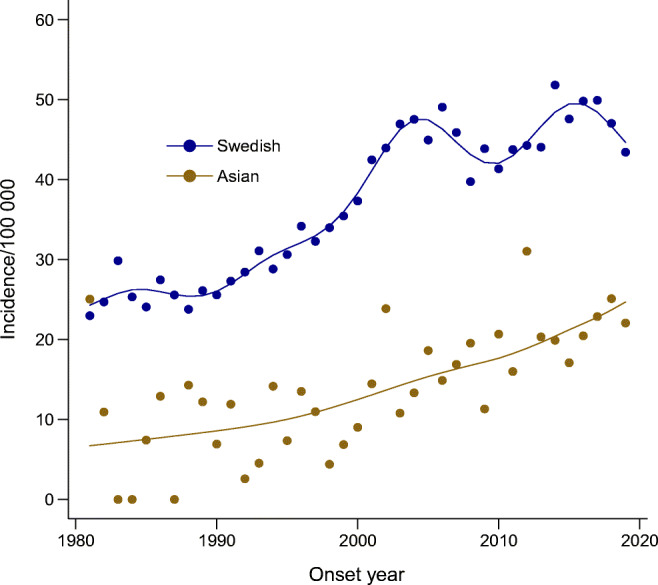
Incidence of type 1 diabetes in children below 15 years of age per 100,000 by calendar years 1980–2019 for subgroups Swedish (i.e. child and both parents born in Sweden) and Asian (i.e. at least one parent born in an Asian country)

To illustrate the effect of genetic traits in different subpopulations, we extracted children with at least one parent born in an Asian country, including the IDF regions of the Middle East and South-East Asia [5]. Among Asian children we observed a steady increase in incidence over the whole study period but at a much lower level than that seen in the Swedish population (Fig. 4).

## Discussion

The present follow-up of the incidence trend of childhood-onset type 1 diabetes in Sweden over the last 40 years shows a prolongation of the levelling off indicated in the early 2000s, although still at a high level of incidence [2]. Similar trends have been observed in some other countries, albeit with shorter observation times [11–13]. The hypothesis that increased immigration to Sweden over the past decades has caused a stabilisation of the incidence rate due to an increased fraction of children with fewer susceptible genetic traits does not seem to explain the development of the incidence trends. Instead, a Swedish subpopulation displayed a similar deceleration in incidence rate, although with some variability. The lower incidence level for children with immigrant parents compared with those with Swedish parents is evidence that genetics may determine the incidence level, whereas the trend is mainly due to environmental risk factors. This is illustrated by the low, but still increasing trend in incidence in children with at least one parent born in an Asian country, that is, with an extremely low trait for type 1 diabetes [21]. This increase in incidence over time after exposure to environmental triggers has also been shown, for example, in Japanese children living in Japan, where a plateau in incidence was indicated after 1992 [22]. As the risk genes may also differ in Asian populations and may respond differently to environmental factors, it would be interesting to follow this low-risk population living in Sweden further.

Of the non-genetic risk factors suggested, lifestyle habits, for example dietary patterns and early growth rate, have been proposed to be triggers and/or accelerators of beta cell destruction in combination with a complex genetic background [7–10]. In Sweden, as in many other countries, the prevalence of childhood obesity increased in the late 1990s [23]. Swedish data based on routine weight–length measurements of schoolchildren, however, indicate that the obesity epidemic reached a plateau during the first decade of the 2000s [24, 25]. Moreover, a recent worldwide pooling of data from population-based studies, analysing trends in BMI and weight over the period 1975–2016 [26], showed that the increase in BMI in children and adolescents has plateaued in many high-income countries during recent years.

Another risk factor for type 1 diabetes in the Swedish setting is maternal BMI, particularly obesity in the first trimester [27]. However, a recent study indicates that there have been lower rates of increase in BMI and obesity prevalence in Swedish women since the early 2000s [28].

These ecological associations that indicate that the incidence trends in childhood-onset diabetes may partly depend on changes in childhood growth and weight are supported by several population-based case–referent studies showing such associations at the individual level [29–33].

A strength of our study is that it is population-based, is nationwide and represents a long period of follow-up and a large number of cases with data collected from reliable sources, both for the overall study population and for the subgroups. Linking individual cases to their parents, and in turn their countries of birth, provides new knowledge of the trends involved. The subpopulation analyses of cases born in Sweden with Swedish parents compared with other groups, and the more specific comparison made with cases with an Asian origin, provide further support for the observed incidence trends owing to the similar patterns of diabetes incidence over time. However, limitations of this study include lack of detailed comparisons of subgroups of other origins, and also the absence of individually linked variables that would support our hypothesis on the importance of changes in growth.

We conclude that, after more than 25 years of rapid increase in the 1980s and 1990s, the incidence of childhood-onset type 1 diabetes has levelled off in Sweden over the last two decades, despite a noted variability in the older age group. This pattern is not dependent on the increased immigration level over the same period. The association with the time trend of obesity in Sweden is of clear interest.

The number of incident cases of this burdensome disease remains high in Sweden and the results indicate that it is important to continue to monitor future developments and intensify efforts to improve lifestyle habits among young children and their families.

## Supplementary Information

ESM 1 (PDF 104 kb)

ESM 2 (XLSX 49 kb)

## Data Availability

The data that support the findings of this study are available from the Swedish National Board of Health and Welfare and Statistics Sweden. Restrictions apply to the availability of these data, which were used under licence for the current study and are not publicly available. Data are, however, available from the authors on reasonable request and with permission of the National Board of Health and Welfare and Statistics Sweden. Incidence data are provided in the ESM (ESM Table 2).

## Abbreviations

GAM: Generalised additive model
SCDR: Swedish Childhood Diabetes Registry

## References

[1] Dahlquist G, Blom L, Holmgren G (1985). The epidemiology of diabetes in Swedish children 0-14 years--a six-year prospective study. Diabetologia.

[2] Berhan Y, Waernbaum I, Lind T, Möllsten A, Dahlquist G (2011). Thirty years of prospective nationwide incidence of childhood type 1 diabetes: the accelerating increase by time tends to level off in Sweden. Diabetes.

[3] DIAMOND Project Group (2006). Incidence and trends of childhood type 1 diabetes worldwide 1990–1999. Diabet Med.

[4] Patterson CC, Harjutsalo V, Rosenbauer J (2019). Trends and cyclical variation in the incidence of childhood type 1 diabetes in 26 European centres in the 25 year period 1989–2013: a multicentre prospective registration study. Diabetologia.

[5] Patterson CC, Karuranga S, Salpea P (2019). Worldwide estimates of incidence, prevalence and mortality of type 1 diabetes in children and adolescents: results from the International Diabetes Federation Diabetes Atlas, 9th edition. Diabetes Res Clin Pract.

[6] Atkinson MA, Eisenbarth GS, Michels AW (2014). Type 1 diabetes. Lancet.

[7] Gale EAM (2002). The rise of childhood type 1 diabetes in the 20th century. Diabetes.

[8] Dahlquist G (2006). Can we slow the rising incidence of childhood-onset autoimmune diabetes? The overload hypothesis. Diabetologia.

[9] Wilkin TJ (2001). The accelerator hypothesis: weight gain as the missing link between type I and type II diabetes. Diabetologia.

[10] Norris JM, Johnson RK, Stene LC (2020). Type 1 diabetes—early life origins and changing epidemiology. Lancet Diabetes Endocrinol.

[11] Skrivarhaug T, Stene LC, Drivvoll AK, Strøm H, Joner G, Norwegian Childhood Diabetes Study Group (2014). Incidence of type 1 diabetes in Norway among children aged 0–14 years between 1989 and 2012: has the incidence stopped rising? Results from the Norwegian Childhood Diabetes Registry. Diabetologia.

[12] Haynes A, Bulsara MK, Jones TW, Davis EA (2018). Incidence of childhood onset type 1 diabetes in Western Australia from 1985 to 2016: evidence for a plateau. Pediatr Diabetes.

[13] Parviainen A, But A, Siljander H, Knip M, Finnish Pediatric Diabetes Register (2020). Decreased incidence of type 1 diabetes in young Finnish children. Diabetes Care.

[14] Dabelea D, Mayer-Davis EJ, Saydah S (2014). Prevalence of type 1 and type 2 Diabetes among children and adolescents from 2001 to 2009. JAMA.

[15] Mayer-Davis EJ, Lawrence JM, Dabelea D (2017). Incidence trends of type 1 and type 2 diabetes among youths, 2002–2012. N Engl J Med.

[16] Population Statistics. In Statistics Sweden. Available from https://www.scb.se/en/finding-statistics/statistics-by-subject-area/population/population-comnpositio/population-statistics/ Accessed 27 Sep 2022

[17] Dahlquist G, Mustonen L (2000). Analysis of 20 years of prospective registration of childhood onset diabetes–time trends and birth cohort effects. Acta Paediatr.

[18] Socialstyrelsen (2022). National prescribed drug register. Available from https://www.socialstyrelsen.se/en/statistics-and-data/registers/register-information/national-prescribed-drug-register/. Accessed 16 Jan 2022

[19] Xia Y, Xie Z, Huang G, Zhou Z (2019). Incidence and trend of type 1 diabetes and the underlying environmental determinants. Diabetes Metab Res Rev.

[20] R Development Core Team (2021) R: the R project for statistical computing. Available from https://www.r-project.org/. Accessed 16 Jan 2022

[21] Park Y (2006). Why is type 1 diabetes uncommon in Asia?. Ann N Y Acad Sci.

[22] Kawasaki E, Matsuura N, Eguchi K (2006). Type 1 diabetes in Japan. Diabetologia.

[23] Mårild S, Bondestam M, Bergström R, Ehnberg S, Hollsing A, Albertsson-Wikland K (2004). Prevalence trends of obesity and overweight among 10-year-old children in western Sweden and relationship with parental body mass index. Acta Paediatr.

[24] Sjöberg A, Lissner L, Albertsson-Wikland K, Mårild S (2008). Recent anthropometric trends among Swedish school children: evidence for decreasing prevalence of overweight in girls. Acta Paediatr.

[25] Lissner L, Sohlström A, Sundblom E, Sjöberg A (2010). Trends in overweight and obesity in Swedish schoolchildren 1999-2005: has the epidemic reached a plateau?. Obes Rev.

[26] Abarca-Gómez L, Abdeen ZA, Hamid ZA (2017). Worldwide trends in body-mass index, underweight, overweight, and obesity from 1975 to 2016: a pooled analysis of 2416 population-based measurement studies in 128·9 million children, adolescents, and adults. Lancet.

[27] Waernbaum I, Dahlquist G, Lind T (2019). Perinatal risk factors for type 1 diabetes revisited: a population-based register study. Diabetologia.

[28] Lundberg CE, Ryd M, Adiels M, Rosengren A, Björck L (2021). Social inequalities and trends in pre-pregnancy body mass index in Swedish women. Sci Rep.

[29] Dahlquist GG, Blom LG, Persson LA, Sandström AI, Wall SG (1990). Dietary factors and the risk of developing insulin dependent diabetes in childhood. BMJ.

[30] Blom L, Persson LÅ, Dahlquist G (1992). A high linear growth is associated with an increased risk of childhood diabetes mellitus. Diabetologia.

[31] Hyppönen E, Virtanen SM, Kenward MG, Knip M, Åkerblom HK (2000). Obesity, increased linear growth, and risk of type 1 diabetes in children. Diabetes Care.

[32] Pundziūtė-Lyckå A, Persson L-Å, Cedermark G (2004). Diet, growth, and the risk for type 1 diabetes in childhood. Diabetes Care.

[33] EURODIAB Substudy 2 Study Group (2002). Rapid early growth is associated with increased risk of childhood type 1 diabetes in various European populations. Diabetes Care.

