# Supplementary material for: The incidence of childhood-onset type 1 diabetes, time trends and association with the population composition in Sweden: a 40 year follow-up
Source: Diabetologia. 2022 Oct 20;66(2):346–53. doi: 10.1007/s00125-022-05816-0 (PMC9807495; doi:10.1007/s00125-022-05816-0)
Supplement: Supplementary file 1 — (PDF 104 kb) [file 125_2022_5816_MOESM1_ESM.pdf]

Electronic supplementary material (ESM)

ESM Table 1. Concordance between the two methods of registration of incident diabetes cases.

	Year of diabetes onset			
	2007	2008	2009	Overall
Method of registration				
Reported by the pediatric clinics (n)	646	634	673	1953
Filled prescriptions of insulin (n)	678	638	695	2011
Difference (n (%))	32 (4.7)	4 (0.6)	22 (3.2)	58 (2.9)

The difference between the two methods of registering new diabetes cases in the SCDR was 2.9% over the three years preceding the switch from reports of cases from the pediatric clinics to reports of filled prescriptions of insulin.

ESM Table 3: Incident cases with person years at risk by age categories and sex for children born in Sweden (*Swedish*) with both parents born in Sweden and the counterpart (*Other countries*), with at least one parent not born in Sweden and overall population.

	Swedish, n= 18 616<br>(person years at risk)	Other countries, n=4 527<br>(person years at risk)	Overall, n=23 143<br>(person years at risk)
Age categories			
0-4 years	3978 (16553844)	958 (5722855)	4936 (22276699)
5-9 years	7 846 (16872533)	1 945 (5685067)	9 791 (22557600)
10-14 years	6 792 (17325607)	1 624 (5590802)	8 416 (22916409)
Sex			
Girls	8 654 (24667851)	2 211 (8317047)	10 865 (32984898)
Boys	9 962 (26084133)	2 316 (8681677)	12 278 (34765810)
